# A Disclaimer

**Published:** 1914-05-09

**Authors:** Edwin L. Ash

**Affiliations:** 56 Seymour Street, Portman Square, W.


*To the Editor of* The Hospital.

### A Disclaimer.

*To the Editor of* The Hospital.

Sir,—The reference to the London Nerve Clinic contained in a letter in the early editions of the *Pall Mall Gazette* to-day (May 5) has been published entirely without my knowledge or consent. On its being brought to my notice I have immediately made suitable protests to the writer of the letter and endeavoured to prevent its further publication.—I am, faithfully yours,

Edwin L. Ash.

56 Seymour Street, Portman Square, W.
May 5, 1914.

